# Risk factors for severe COVID-19 outcomes in LATAM countries in the post-vaccination era: an analysis of national surveillance data in Argentina, Brazil, Colombia, and Mexico

**DOI:** 10.7189/jogh.15.04141

**Published:** 2025-04-28

**Authors:** Guilherme Silva Julian, Júlia Spinardi, Melissa Díaz, Diana Buitrago Ospina, Nohemi Caballero, Vinicius Goularte-Silva, Moe H Kyaw

**Affiliations:** 1 Pfizer, Evidence Generation Medical Affairs, Brazil; 2 Pfizer, Vaccines Medical Affairs, Brazil; 3 Faculdade de Ciências Médicas da Santa Casa de São Paulo, São Paulo, Brazil; 4 IQVIA, Real World Insights, Bogotá, Colombia; 5 IQVIA, Real World Insights, São Paulo, Brazil; 6 Pfizer, Vaccines, Scientific Affairs, Collegeville, Pennsylvania, USA

## Abstract

**Background:**

Identifying cases at higher risk for severe COVID-19 outcomes is essential for tailoring interventions for prevention and treatment. We aimed to determine the factors related to hospitalisation, intensive care unit (ICU) admission, use of ventilatory support, and mortality in four Latin American countries.

**Methods:**

We conducted a retrospective study using national COVID-19 surveillance databases from Argentina, Brazil, Colombia, and Mexico, covering the period from January 2021 to December 2022. We used multivariate logistic regression models to identify factors associated with hospitalisation, ICU admission, ventilatory support, and death, adjusting for confounding variables.

**Results:**

We included 34 955 384 confirmed cases in the analysis. Age and sex were significantly associated with increased odds of all outcomes. For hospitalisation, in cases aged >85 years, the odds ratio (OR) for hospitalisation ranged from 26.46 (95% confidence interval (CI) = 25.67–27.28) in Mexico to 2763.87 (95% CI = 2644.40–2888.73) in Brazil, and for males, it ranged from 1.42 (95% CI = 1.41–1.43) in Colombia to 1.77 (95% CI = 1.76–1.78) in Brazil. Indigenous race was significantly associated with higher odds of hospitalisation (ORs ranging from 1.26 to 1.98) and death (ORs ranging from 1.05 to 1.84). The number of comorbidities reported was related to increased odds of severe outcomes and varied across countries. The odds of death for cases with zero vaccine doses were significantly higher (ORs ranging from 1.72 to 31.73) compared to cases with two doses. Similarly, the odds of death for cases with one dose were significantly higher (ORs ranging from 1.73 to 7.00) compared to cases with two doses.

**Conclusions:**

Even in a post-vaccine implementation scenario, individual factors such as age, gender, comorbidities, and race still pose a risk to severe COVID-19, which demands tailoring public health strategies for prevention and treatment.

The Latin American (LATAM) region has been particularly affected by COVID-19 [1,2]. Despite constituting approximately 8.4% of the world’s population, by February 2022, it had already accounted for almost 66 million infections (15% of the global total) and 1.65 million deaths (28% of the global total) [2]. From 1 January 2020 to 9 January 2024, 193.20 million COVID-19 cases were reported in the Americas, with 13.67 million requiring hospital admission and 2.98 million resulting in deaths [3]. Brazil, Argentina, Mexico, and Colombia are among the countries with the highest number of accumulated cases in the region. Up to 19 December 2023, Brazil reported 37 519 960 accumulated confirmed cases; Argentina followed with 10 074 137, Mexico with 7 702 582, and Colombia with 6 384 551 [3].

In early March 2021, Colombia became the first country in LATAM to receive mRNA vaccines through the COVID-19 Vaccines Global Access (COVAX) programme [4]. mRNA, adenoviral vector-based, and inactivated virus vaccines have largely been recommended for the primary series in adults across LATAM [5]. Subsequently, mRNA vaccines were the preferred platform for administering booster doses [5].

Vaccine deployment in the region has been significant. As of 5 January 2024, 83.5 per 100 individuals in Argentina had received a complete primary schedule [6]. Brazil, Colombia, and Mexico reported rates of 80.8, 72.6, and 62.4 per 100 individuals with a complete primary schedule, respectively [6].

Despite significant reductions in severe outcomes with the advent of COVID-19 vaccination, severe complications persist. As reported by the Pan American Health Organization (PAHO), in Brazil, 33.72% of hospitalised cases are admitted to the intensive care unit (ICU), while 30.28% have a fatal outcome [3]. Argentina reports 18.17% of hospitalised cases requiring ICU admission, with a 46% fatality rate, compared to 3.36% and 39.25% in Mexico, respectively [3].

Identifying cases at higher risk for severe outcomes is essential for tailoring targeted interventions for prevention and timely treatment. In this retrospective nationwide database study, we used COVID-19 surveillance data from Argentina, Brazil, Colombia, and Mexico to analyse the factors related to severe outcomes (hospitalisation, ICU admission, utilisation of ventilatory support, and mortality) during the period when the countries had already started to implement their COVID-19 vaccination plans.

## METHODS

We retrieved data on all confirmed cases reported to COVID-19 national surveillance databases in Argentina, Brazil, Colombia, and Mexico between January 2021 and December 2022. This period saw the introduction and implementation of COVID-19 vaccines in these countries, as well as the simultaneous circulation of multiple SARS-CoV-2 variants. In 2021, the 20J Gamma V3 variant predominated in Brazil (in Q1, Q2, and Q3), Mexico (in Q3 and Q4), and Argentina (in Q2 and Q3). The same year, the 21H Mu variant was predominant in Colombia from Q1 to Q3. In 2022, the Omicron variant was present in all countries [7].

### Data sources

We used the COVID-19 surveillance dataset from Argentina [8], the Flu syndrome notifications database (*Sistema de Informação de Vigilância Sentinela de Sindrome Gripal* (*eSUS-Notifica SG*)) and the Influenza Epidemiological Surveillance Information System (*Sistema de Informação de Vigilância Epidemiológica da Gripe* (*SIVEP-Flu*)) database through the OpenDATASUS platform from Brazil [9,10], the COVID module and the Individual Registry of Health Services Provision module (*Registro Individual de Prestación de Servicios de Salud* (*RIPS*)) from the Information System for Social Protection (*Sistema de Información para la Protección Social* (*SISPRO*)) database from Colombia [11], and the COVID-19 database from Mexico, which is consolidated by the Epidemiological Surveillance System for Viral Respiratory Diseases [12] (Figure S1 and Table S1 in the **Online Supplementary Document**).

For Brazil, Mexico, and Colombia, we retrieved data on confirmed cases between January 2021 and December 2022. For Argentina, given data availability, we retrieved case-level data on all confirmed COVID-19 cases from January 2021 to June 2022. Data regarding hospitalisation in the general ward, ICU admission, ventilatory support, and death were available for all countries, except for Colombia, where data about ICU admission were not available. Databases from all countries contained information about age and sex; those from Brazil, Colombia, and Mexico further included information about race and comorbidities; and those from Brazil and Colombia further included information about COVID-19 vaccination status (Table S2 in the **Online Supplementary Document**).

### Outcomes and variables

We evaluated the following outcomes among confirmed COVID-19 cases: hospitalisation in the general ward, ICU admission, use of ventilatory support (defined as the assistance to breathing provided by mechanical means), and mortality. We also analysed the year of case occurrence and sociodemographic variables, including age, sex, and race. Because the comorbidities reported in each country’s database varied, we analysed the number of comorbidities reported according to the comorbidities available for each country. Finally, we examined the vaccination status, when available, according to the number of doses administered.

### Statistical methods

We conducted descriptive analyses to summarise the sociodemographic characteristics (age, sex, and race), comorbidities, and vaccination status of confirmed COVID-19 cases in each country. We computed frequencies and percentages for categorical variables and medians with interquartile ranges or means with standard deviations for quantitative variables. We conducted bivariate analyses to explore the associations between these variables and clinical outcomes. We also performed the chi-squared test to assess the association between categorical variables and hospitalisation, ventilatory support, ICU admission, or fatal outcome. Lastly, we carried out multivariate logistic regression models to identify factors associated with each outcome, adjusting for confounding variables. We reported odds ratios (ORs) and 95% confidence intervals (CIs) for the predictive variables age group, sex, race, number of comorbidities, year of case occurrence, and vaccination status (when available). Statistical analyses were conducted in Python, version 3.8.10 (Python Software Foundation, Wilmington, Delaware, USA).

## RESULTS

We analysed 34 955 384 confirmed cases (Figure S2 in the **Online Supplementary Document**), with 7 131 436 (20.4%) being from Argentina, 17 751 803 (50.8%) from Brazil, 4 644 148 (13.3%) from Colombia, and 5 427 997 (15.5%) from Mexico. In total, 2 006 458 (5.7%) cases required hospitalisation in the general ward, 997 901 (2.9%) used ventilatory support, and 769 161 (2.2%) died (**Table 1**). Furthermore, 500 167 (1.7%) cases from Argentina, Brazil, and Mexico required ICU admission. Data for this outcome were not available for Colombia.

**Table 1:** Characteristics of included cases across all countries and according to clinical outcomes*

		Hospitalisation		Ventilatory support		ICU admission		Fatal outcome	
	**Total confirmed cases**	**Yes**	**No**	**Yes**	**No**	**Yes**	**No**	**Yes**	**No**
**Total number**	34 955 384	2 006 458	32 948 926	997 901	17 357 952	500 167	17 753 422	769 161	34 186 223
**Age in years**									
0–4	547 037 (1.6)	39 663 (2.0)	507 374 (1.5)	10 815 (1.1)	341 014 (2.0)	6244 (1.2)	342 442 (1.9)	1678 (0.2)	545 359 (1.6)
5–17	2 316 207 (6.6)	26 366 (1.3)	2 289 841 (6.9)	6145 (0.6)	1 219 945 (7.0)	3753 (0.8)	1 220 978 (6.9)	1634 (0.2)	2 314 573 (6.8)
18–29	7 597 465 (21.7)	93 853 (4.7)	7 503 612 (22.8)	35 801 (3.6)	3 461 129 (19.9)	16 335 (3.3)	3 472 304 (19.6)	11 785 (1.5)	7 585 680 (22.2)
30–39	7 568 883 (21.7)	198 609 (9.9)	7 370 274 (22.4)	99 314 (10.0)	3 596 617 (20.7)	43 142 (8.6)	3 637 950 (20.5)	35 453 (4.6)	7 533 430 (22.0)
40–49	6 623 829 (18.9)	294 099 (14.7)	6 329 730 (19.2)	156 822 (15.7)	3 346 659 (19.3)	71 134 (14.2)	3 415 000 (19.2)	73 566 (9.6)	6 550 263 (19.2)
50–64	4 178 036 (12.0)	571 451 (28.5)	3 606 585 (10.9)	301 266 (30.2)	1 007 552 (5.8)	149 929 (30.0)	1 129 390 (6.4)	210 924 (27.4)	3 967 112 (11.6)
65–74	1 198 109 (3.4)	363 956 (18.1)	834 153 (2.5)	184 772 (18.5)	164 601 (0.9)	101 956 (20.4)	233 116 (1.3)	183 804 (23.9)	1 014 305 (3.0)
75–84	620 487 (1.8)	268 963 (13.4)	351 524 (1.1)	130 709 (13.1)	121 984 (0.7)	71 482 (14.3)	171 913 (1.0)	153 880 (20.0)	466 607 (1.4)
≥85	271 749 (0.8)	148 848 (7.4)	122 901 (0.4)	71 963 (7.2)	66 508 (0.4)	35 953 (7.2)	98 331 (0.6)	96 294 (12.5)	175 455 (0.5)
Missing	4 033 582 (11.5)	650 (0.0)	4 032 932 (12.5)	294 (0.0)	4 031 943 (23.2)	239 (0.0)	4 031 998 (22.7)	143 (0.0)	4 033 439 (11.8)
**Sex**									
Female	18 950 032 (54.2)	896 279 (44.7)	18 053 753 (54.8)	441 456 (44.2)	9 664 250 (55.7)	215 665 (43.1)	9 841 849 (55.4)	331 216 (43.1)	18 618 816 (54.5)
Male	15 973 227 (45.7)	1 108 942 (55.3)	14 864 285 (45.1)	556 295 (55.7)	7 692 176 (44.3)	284 280 (56.8)	7 910 216 (44.6)	437 262 (56.8)	15 535 965 (45.4)
Missing	32 125 (0.1)	1237 (0.1)	30 888 (0.1)	150 (0.0)	1526 (0.0)	222 (0.0)	1357 (0.0)	683 (0.1)	31 442 (0.1)
**Race**									
Other	13 932 518 (50.1)	1 098 389 (58.5)	12 834 129 (49.5)	796 655 (81.4)	13 052 938 (75.5)	365 710 (78.0)	13 482 618 (76.4)	478 364 (69.4)	13 988 631 (51.6)
Indigenous	9 785 207 (35.2)	553 264 (29.5)	9 231 943 (35.6)	34 516 (3.5)	453 818 (2.6)	24 229 (5.2)	363 106 (2.1)	245 681 (35.6)	9 541 210 (35.2)
Not specified	227 499 (0.8)	10 938 (0.6)	216 561 (0.8)	1296 (0.1)	9533 (0.1)	1334 (0.3)	9495 (0.1)	4077 (0.6)	223 422 (0.8)
Missing	3 878 724 (13.9)	215 494 (11.5)	3 663 230 (14.1)	146 706 (15.0)	3 732 018 (21.6)	77 658 (16.6)	3 801 066 (21.5)	73 495 (10.7)	3 912 502 (14.4)
**COVID-19 vaccination status†**									
Booster	1 591 585 (7.1)	85 375 (5.7)	1 506 210 (7.2)	52 957 (5.6)	1 538 628 (9.2)	26 079 (5.9)	1 565 506 (9.0)	35 952 (6.8)	1 622 173 (7.4)
Two doses	7 267 049 (32.4)	268 504 (17.9)	6 998 545 (33.5)	124 236 (13.2)	3 709 447 (22.1)	61 926 (14.0)	3 771 757 (21.8)	108 580 (20.4)	7 283 560 (33.3)
One dose	1 710 597 (7.6)	144 556 (9.6)	1 566 041 (7.5)	93 724 (9.9)	1 125 223 (6.7)	43 365 (9.8)	1 175 582 (6.8)	66 469 (12.5)	1 693 128 (7.7)
Zero doses	11 826 720 (52.8)	1 004 504 (66.8)	10 822 216 (51.8)	673 129 (71.3)	10 434 459 (62.1)	311 989 (70.4)	10 795 599 (62.4)	432 318 (81.4)	11 797 205 (54.0)

*Values are presented as numbers (%).

†There were no missing data.

**Figure 1:**
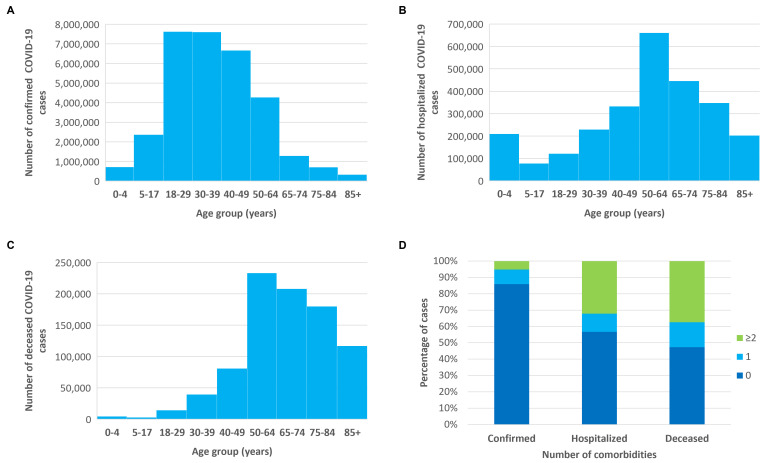
Age and comorbidities distribution of COVID-19 confirmed, hospitalised, and deceased cases. **Panel A.** Number of confirmed cases by age. **Panel B.** Number of hospitalised cases by age. **Panel C.** Number of deceased cases by age. **Panel D.** Percentage of cases by the number of comorbidities.

Considering all countries, most confirmed COVID-19 cases were aged 18–49 years (62.3%) (**Figure 1**, Panel A). Overall, COVID-19 cases requiring hospitalisation in the general ward were predominantly adults, with a peak in cases aged 50–64 years and the largest proportion of deaths occurring in cases aged 50 to >85 years (83.8%) (**Figure 1****,** Panels B and C). As for sex most confirmed COVID-19 cases were female (54.2%), whereas males constituted the majority among hospitalised cases (55.3%), cases requiring ventilatory support (55.7%), ICU admission (56.8%), or those who were deceased (56.8%) (**Table 1**). Including data from Colombia, Brazil, and Mexico, the proportion of cases with two or more comorbidities was higher among deceased (42.9%) and hospitalised (36.0%) cases compared with confirmed cases (4.0%) (**Figure 1**, Panel D).

### Hospitalisation

**Figure 2:**
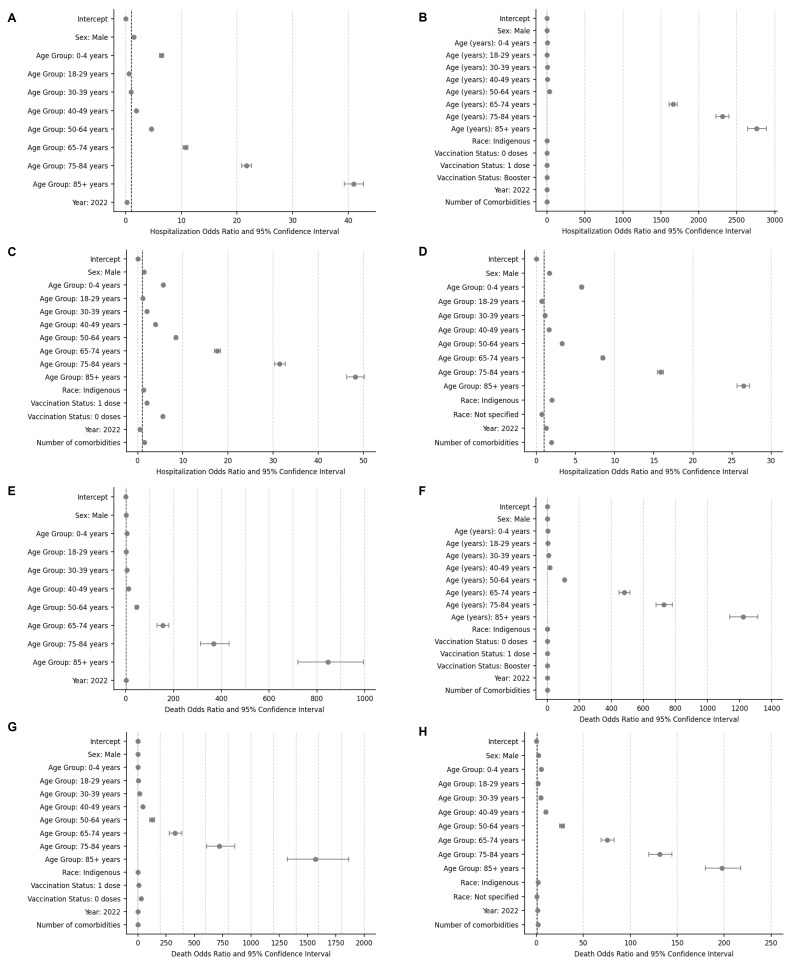
Factors related to hospitalisation and death in the general ward among COVID-19 confirmed cases across countries. **Panel A.** Hospitaliasation in Argentina. **Panel B.** Hospitalisation in Brazil. **Panel C.** Hospitalisation in Colombia. **Panel D.** Hospitalisation in Mexico. **Panel E.** Death in Argentina. **Panel F.** Death in Brazil. **Panel G.** Death in Colombia. **Panel H.** Death in Mexico.

In Argentina, older age was associated with the probability of requiring hospitalisation in the general ward. The odds of hospitalisation increased significantly in cases >40 years, with a peak among cases >85 years (OR = 40.97; 95% CI = 39.26–42.76). In addition, males were more likely to require hospitalisation compared to females (OR = 1.45; 95% CI = 1.43–1.46) (**Figure 2**, Panel A). Similarly, in Brazil, Colombia, and Mexico, the probability of hospitalisation increased in the >65 age group and males (**Figure 2**, Panels B–D). Except for Mexico, the odds of hospitalisation in the general ward were lower in 2022 compared to 2021, ranging from 0.14 (95% CI = 0.14–0.14) in Brazil to 0.49 (95% CI = 0.48–0.49) in Colombia. In Mexico, the odds were higher in 2022 (OR = 1.22; 95% CI = 1.21–1.23).

According to the COVID-19 vaccination status, in Brazil, cases with one dose of a COVID-19 vaccine were more likely to require hospitalisation compared with those with two doses (OR = 2.21; 95% CI = 2.18–2.25). Each additional comorbidity reported increased the chance of requiring hospitalisation on average by 1.75 (95% CI = 1.75–1.75).

In Colombia, cases of indigenous race had a higher chance of requiring hospitalisation than those of other races (OR = 1.26; 95% CI = 1.20–1.31). When considering vaccination status, cases who received one (OR = 2.03; 95% CI = 2.00–2.06) or zero (OR = 5.53; 95% CI = 5.47–5.59) doses had higher odds of requiring hospitalisation compared with those who received two doses. As for comorbidities, for each additional comorbidity reported, there was an increase in the odds of requiring hospitalisation (OR = 1.47; 95% CI = 1.45–1.48).

In Mexico, cases of indigenous race (OR = 1.97; 95% CI = 1.91–2.04) and cases with a higher number of comorbidities (OR = 1.93, 95% CI = 1.92–1.94) had a higher probability of hospitalisation.

### ICU admission

**Figure 3:**
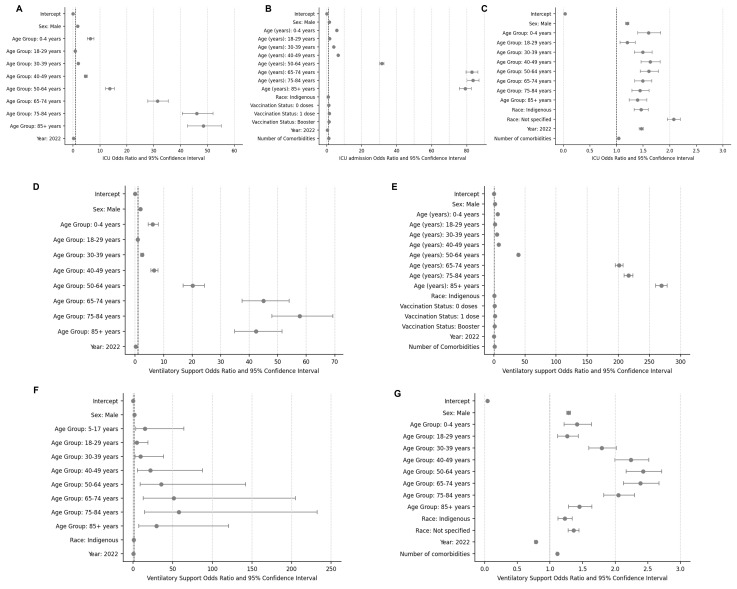
Factors related to ICU admission and the use of ventilatory support among COVID-19 confirmed cases across countries. **Panel A.** ICU admission in Argentina. **Panel B.** ICU admission in Brazil. **Panel C.** ICU admission in Mexico. **Panel D.** Use of ventilatory support in Argentina. **Panel E.** Use of ventilatory support in Brazil. **Panel F.** Use of ventilatory support in Colombia. **Panel G.** Use of ventilatory support in Mexico. ICU – intensive care unit

In Argentina, the odds of ICU admission were higher in cases aged 0–4 and >30 years, compared to those aged 5–17 years (**Figure 3**, Panel A), with the highest odds observed in cases >85 years (OR = 48.45; 95% CI = 42.61–55.10). In Brazil and Mexico, the odds of ICU admission were higher across all age groups compared to those aged 5–17 years, peaking in cases 75–84 years in Brazil (OR = 83.48; 95% CI = 80.10–87.01) and in those aged 40–49 in Mexico (OR = 1.64; 95% CI = 1.46–1.82) (**Figure 3**, Panels B and C). Males were more likely to be admitted to the ICU than females in Argentina (OR = 1.71), Brazil (OR = 1.48), Mexico (OR = 1.20).

The odds of ICU admission were lower in 2022 compared to 2021 in Argentina (OR = 0.18; 95% CI = 0.17–0.19) and Brazil (OR = 0.32; 95% CI = 0.32–0.33). In Mexico, the odds were higher in 2022 (OR = 1.47; 95% CI = 1.43–1.50).

In Brazil, when considering vaccination status, the odds of ICU admission were higher in cases with zero (OR = 1.17; 95% CI = 1.15–1.18) or one (OR = 1.41; 95% CI = 1.38–1.43) doses compared to those with two doses.

In Mexico, cases of indigenous race had higher odds of ICU admission compared with other races (OR = 1.45; 95% CI = 1.33–1.59). Finally, as the number of comorbidities reported increased, the odds of ICU admission increased on average by 1.04 (95% CI = 1.02–1.05).

### Ventilatory support

Across all countries, male cases were more likely to use ventilatory support compared with females, and the odds of requiring ventilatory support were higher in 2022 than in 2021 (**Figure 3**, Panels D–G). In Brazil, Colombia, and Mexico, the odds of using ventilatory support were higher in all age groups compared to those aged 5–17 years. The age groups with the highest odds of using ventilatory support were those aged >85 years in Brazil (OR = 268.86; 95% CI = 259.78–278.25), those aged 75–84 in Colombia (OR = 57.67; 95% CI = 12.72–205.03), and those aged 50–64 in Mexico (OR = 2.4; 95% CI = 2.17–2.72). In Brazil, cases with one vaccine dose had higher chances of using ventilatory support compared with those with two doses (OR = 1.88; 95% CI = 1.85–1.91).

### Death

Among COVID-19 confirmed cases in Argentina, Brazil, Colombia, and Mexico, male sex and older age were associated with a higher probability of death (**Figure 2**, Panels E–H). Individuals aged >85 years had the highest odds of death across all countries, with Brazil exhibiting the highest odds among these cases (OR = 1222.36; 95% CI = 1138.04–1312.90). In all countries except for Mexico, cases registered in 2022 were more likely to have a fatal outcome compared to those registered in 2021. In Brazil and Mexico, indigenous cases were also more likely to have a fatal outcome compared with cases of another race.

In Brazil, the odds of death among COVID-19 cases were higher in those with zero (OR = 1.72; 95% CI = 1.70–1.79) or one (OR = 1.73; 95% CI = 1.70–1.76) dose of a COVID-19 vaccine than in those with two doses. In addition, cases with a booster dose had a lower probability of dying compared to those with two doses (OR = 0.82; 95% CI = 0.80–0.84). These findings were also observed in Colombia, where cases without any vaccine dose had a 32-fold (95% CI = 31.14–32.34) higher possibility of death compared with cases with two doses, and cases with one dose had seven times (95% CI = 6.82–7.17) the chance of those with two doses.

In Brazil, Colombia, and Mexico, the odds of death increased with each additional comorbidity reported. For instance, in Colombia, the odds of death increased on average by 1.77 for each additional comorbidity reported (95% CI = 1.75–1.79).

In Brazil, cases with decompensated chronic respiratory diseases (OR = 2.86; 95% CI = 2.68–3.05) and neoplasia (OR = 2.71; 95% CI = 2.57–2.86) were more likely to die (Table S3 in the **Online Supplementary Document**). In Colombia, cases with arthritis were 2.55 times (95% CI = 2.35–2.78) more likely to die compared with those without the disease, while those with orphan diseases (OR = 2.39; 95% CI = 2.14–2.68), HIV infection (OR = 2.22; 95% CI = 1.96–2.51), diabetes mellitus (OR = 1.82; 95% CI = 1.78–1.87), hypertension (OR = 1.73; 95% CI = 1.70–1.77), and cancer (OR = 1.46; 95% CI = 1.39–1.53) also had a higher probability of dying (Table S4 in the **Online Supplementary Document**). In Mexico, cases with obesity (OR = 1.65; 95% CI = 1.60–1.69), immunosuppression (OR = 1.20; 95% CI = 1.12–1.29), hypertension (OR = 1.09; 95% CI = 1.06–1.11), and cardiovascular disease (OR = 1.07; 95% CI = 1.01–1.13) were more likely to have a fatal outcome (Table S5 in the **Online Supplementary Document**).

## DISCUSSION

In this study, we analysed the factors related to hospitalisation, ICU admission, utilisation of ventilatory support, and mortality among confirmed COVID-19 cases from Argentina, Brazil, Colombia, and Mexico in the context of COVID-19 vaccination implementation. Our results showed that older age, male sex, indigenous race, and a higher number of comorbidities were associated with severe outcomes. Being unvaccinated or receiving only one vaccine dose was also associated with poorer outcomes compared to receiving two doses during the study period. Moreover, having a vaccine booster was associated with additional protection against death.

While multiple previous studies have reported that older age correlates with an increased risk of severe COVID-19 outcomes, particularly in cases who are ≥65 years [13], our data suggest that younger thresholds may need to be considered for LATAM. Although older age was associated with an increased possibility of severe COVID-19 outcomes in all countries studied, in Colombia, for instance, the OR for mortality among COVID-19 confirmed cases began to rise as early as the age of 18. Previous research conducted in LATAM reported a trend of an increased proportion of deaths occurring in cases who are <60 years in multiple countries in 2021 [2]. Studies conducted before the introduction of COVID-19 vaccines suggested that this trend could be attributed to delays in implementing containment measures, disproportionately affecting younger individuals who were more likely to commute regularly for work or school [14].

Males had a higher OR for hospitalisation, ICU admission, use of ventilatory support, and death across countries. Our results are consistent with those of a retrospective cohort that evaluated risk factors for mortality among cases hospitalised for COVID-19 in Brazil in 2021, which reported that women were less likely to have a fatal outcome [15]. Male sex has been established as a factor related to poor COVID-19 outcomes in previous studies globally [16–18]. As for the biological reason for this association, previous research posited that the reduction in testosterone levels in ageing men has been associated with increased proinflammatory cytokine levels, possibly contributing to worse COVID-19 outcomes in older men [18]. Additionally, sex differences in disease outcomes may also be linked to oestrogen-induced decreased expression of angiotensin-converting enzyme 2, the functional receptor of SARS-CoV-2 [17,19]. Behavioural factors may also contribute to observed sex differences, as previous studies have identified that risk perception is a stronger driver of risk behaviour for men than women, who tend to adopt safe measures to protect themselves and others [20]. Women generally adopt safer measures to protect themselves and others, which may contribute to their lower rates of severe outcomes. This suggests that public health messaging and interventions for COVID-19 and future emerging diseases should be tailored to address these gender-specific differences in risk perception and behaviour to increase compliance with recommended protective measures.

In Brazil and Colombia, unvaccinated cases and those with only one vaccine dose had a higher possibility of presenting all severe outcomes. Additionally, in Brazil, receiving a booster dose was associated with lower odds of death. Our results are consistent with previous findings in the region. A real-world study about the effectiveness of vaccines showed that protection against severe COVID-19 outcomes increased when booster doses were administered [21]. Moreover, studies conducted outside LATAM have also highlighted the importance of booster doses to prevent severe COVID-19 outcomes. A case-control study in the USA estimated that vaccine effectiveness was higher after receiving one or two booster doses compared with a primary series alone [22]. Our results, built upon existing evidence, emphasise the importance of completing vaccination schedules and administering booster doses to enhance and sustain immunity against evolving variants, thereby preventing the risk of severe outcomes. Additionally, in LATAM, multiple vaccine platforms, including mRNA, adenoviral vector-based, and inactivated virus vaccines, were implemented. The differences in vaccine efficacy associated with these platforms could have implications for the outcomes observed in our study; however, this type of analysis was not possible due to the intrinsic limitations of the databases analysed. Future research should consider the impact of these different vaccine types when evaluating vaccine effectiveness and policy recommendations for vaccination campaigns.

This increase can likely be attributed to the predominance of the Omicron variant in 2022, which is generally associated with less severe outcomes, and the widespread implementation of vaccination campaigns. For instance, the Omicron variant was associated with a lower risk of hospitalisation, ICU admission, oxygen therapy, ventilation, and death compared to the Delta variant [23,24]. However, in this study, we did not directly analyse the impact of the predominant COVID-19 variants circulating during the study period on the observed outcomes.

We identified indigenous race as a factor associated with severe outcomes across the countries analysed, which is estimated to represent 9.8% of the regional population in LATAM [25]. The COVID-19 pandemic intensified the existing challenges faced by indigenous communities, including difficulties in accessing healthcare services, poverty, mobility restrictions, vulnerability, and insecurity, thereby heightening their susceptibility to adverse outcomes [25]. Moreover, vaccine implementation among indigenous populations presents an additional challenge because cultural practices and beliefs may influence acceptance rates. For instance, as reported by the World Health Organization, in Colombia, there was a reluctance among indigenous populations to receive COVID-19 vaccines based on cultural beliefs. Tailored communication strategies aimed at indigenous communities have been implemented to promote vaccine acceptance [26]. Further research examining the association between indigenous vulnerabilities and severe outcomes is required to develop targeted interventions that effectively improve outcomes among these communities within the region. Historically, indigenous groups have been disproportionately affected by emerging infectious diseases, as evidenced during the H1N1 influenza pandemic in 2009, where indigenous populations experienced higher morbidity and mortality rates in Brazil [27].

We found that as the number of reported comorbidities increased, the probability of severe COVID-19 outcomes increased across countries. Multimorbidity, the presence of two or more long-term health conditions, has been reported to have an impact on distinct phases of COVID-19 [28]. In LATAM and the Caribbean, the prevalence of multimorbidity is estimated to be approximately 37% [29]. This poses a significant challenge to multiple health systems in the region as the number of patients with coexisting diseases continues to grow, aligned with the ageing of the population [30]. We identified that decompensated chronic respiratory diseases, diabetes, hypertension, immunosuppression, orphan diseases, and cancer were associated with a higher risk of death among COVID-19 cases. These conditions have been previously recognised for their impact on COVID-19 severity, with underlying mechanisms differing across pathologies, largely involving an impaired immune response [31]. Considering the role of multimorbidity in COVID-19 and health outcomes in general [32,33], tailored policy strategies that consider multimorbidity are needed to effectively mitigate the impact of COVID-19 and enhance emerging diseases preparedness.

This study has some limitations that may impact its outcomes and conclusions. One limitation is the reporting of ethnicity and race data across the national databases of LATAM countries. Data on these variables are not consistently recorded, leading to a high proportion of missing information. Furthermore, the categorisation of race and ethnicity varies significantly between countries, which complicates direct comparisons. To mitigate this issue, we recategorised data to allow for comparisons between indigenous and other ethnic groups, but this approach may oversimplify the complex racial dynamics within each country. Further, the variables recorded in each country’s database varied, including differences in the information about comorbidities. To address this, we analysed this variable according to the number of comorbidities reported to enable cross-country comparisons, providing an estimate of the impact of multimorbidity on COVID-19 outcomes. Additionally, the retrospective nature of the study and reliance on secondary data may introduce information bias due to missing data or incomplete reporting. To address this issue, we reported all missing information and did not conduct data imputation. However, our study benefits from a large sample size, as well as a multinational, real-world perspective, offering valuable insights for policymakers and clinicians. Moving forward, efforts to address data discrepancies and enhance the quality of surveillance databases will be essential for improving the reliability and validity of future studies in the field.

## CONCLUSIONS

Our findings highlight that even in a post-vaccine implementation scenario, some individuals’ factors, such as age, gender, comorbidities, and race, are still related to a higher risk of severe COVID-19, which demands the tailoring of public health strategies for prevention and treatment. In this context, data from this study can support decision-making and policy development considering local-regional realities.

## Additional material


Online Supplementary Document
