# Supplementary material for: Risk factors for severe COVID-19 outcomes in LATAM countries in the post-vaccination era: an analysis of national surveillance data in Argentina, Brazil, Colombia, and Mexico
Source: J Glob Health. 2025 Apr 28;15:04141. doi: 10.7189/jogh.15.04141 (PMC12035972; doi:10.7189/jogh.15.04141)

Supplement to: Julian GS, Spinardi J, Díaz M, Buitrago D, Caballero N, Goularte-Silva V, Kyaw M. Risk factors for severe COVID-19 outcomes in LATAM countries in the post-vaccination era: an analysis of national surveillance data in Argentina, Brazil, Colombia, and Mexico. J Glob Health. 2025;15:04141.

Table of contents

Supplementary Table S1. Procedure codes for ventilatory support used in Colombia. 2

Supplementary Table S2. Variables definitions for each country 3

Supplementary Table S3. Multivariate Logistic regression for fatal outcome among COVID-19 confirmed cases in Brazil including reported comorbidities. 4

Supplementary Table S4. Multivariate Logistic regression for fatal outcome among COVID-19 confirmed cases in Colombia including reported comorbidities. 5

Supplementary Table S5. Multivariate Logistic regression for fatal outcome among COVID-19 confirmed cases in Mexico including reported comorbidities. 6

Supplementary Figure S1. Data sources and variables across countries. 7

Supplementary Figure S2. Flowchart of included cases. 9

Supplementary Table S1. Procedure codes for ventilatory support used in Colombia.

Code	Procedure
**9390**	**CONTINUOUS POSITIVE PRESSURE BREATHING**
939000	CONTINUOUS POSITIVE PRESSURE BREATHING SOD
**9391**	**INTERMITTENT POSITIVE PRESSURE BREATHING**
939100	INTERMITTENT POSITIVE PRESSURE BREATH SOD
**9601**	**NASOPHARYNGEAL AIRWAY INSERTION**
960100	SOD NASOPHARYNGEAL AIRWAY INSERTION
**9602**	**OROPHARYNGEAL AIRWAY INSERTION**
960200	OROPHARYNGEAL AIRWAY INSERTION SOD
**9603**	**INSERTION OF OBTURATED ESOPHAGEAL AIRWAY**
960300	SOD ESOPHAGEAL OBTURATED AIRWAY INSERTION
**9604**	**ENDOTRACHEAL TUBE INSERTION**
960401	ENDOTRACHEAL TUBE INSERTION WITH RETROGRADE TECHNIQUE
960402	DOUBLE LAMP ENDOTRACHEAL TUBE INSERTION
960403	ENDOTRACHEAL TUBE INSERTION WITH SELECTIVE BLOCKER
960404	INSERTION OF ENDOTRACHEAL TUBE WITH LUMINOUS PROBE
960405	TRANSTRACHEAL JET CANNULA INSERTION
960406	INSERTION OF ENDOTRACHEAL TUBE UNDER ENDOSCOPIC VISION
**9605**	**OTHER RESPIRATORY TRACT INTUBATION**

Supplementary Table S2. Variables definitions for each country

Definition	Brazil	Mexico	Colombia	Argentina
**Confirmed COVID-19**	Laboratorial, regardless of whether the case has a clinical-epidemiological, clinical and/or clinical-image confirmation	Laboratory sample or antigenic test positive for SARS-CoV-2, regardless of whether the case has a clinical-epidemiological association	Laboratory sample or antigenic test positive for SARS-CoV-2	Laboratory test, clinical-epidemiological criteria
**ICU admission**	Variable *UCI* in the database	Variable *UCI* in the database	Not available	Determined by: Variable '*cuidado_intensivo*' in the database
**Ventilatory support**	Ventilatory support determined by the variable *SUPORTE VENTILATÓRIO* in the database	Ventilatory support determined by variable *INTUBADO* in the database	Ventilatory support determined by linkage with RIPS database	Ventilatory support determined by variable *'asistencia_respiratoria_mecanica'* in the database
**Hospital admission**	Hospital admission determined by the variable *HOSPITAL* in the database	Hospital admission determined by the variable *TIPO_PACIENTE*	Hospital admission determined by the variables *Paciente Hospitalizado.Indicador* and *Fecha de Hospitalizacion*	Hospital admission determined by the variable *FECHA INTERNACION*
**Vaccination status**	Vaccination status determined by the variable VACINA_COV	Not available	Vaccination status determined by the variable *Esquema Aplicacion*	Not available
**Death**	Death determined by the option OBITO in the variable EVOLUCAO	Death determined by the completion of the variable FECHA_DEF	Death determined by the option FALLECIDO in the variable RECUPERADO	Death determined by the variable FALLECIDO

Supplementary Table S3. Multivariate Logistic regression for fatal outcome among COVID-19 confirmed cases in Brazil including reported comorbidities.

Characteristic	Brazil		
	**OR**	**95% CI**	
**Age (years)**			
0-4	0.65	0.59	0.72
5-17	Ref		
18-29	1.63	1.50	1.76
30-39	2.14	1.99	2.31
40-49	3.09	2.87	3.33
50-64	5.41	5.03	5.82
65-74	10.11	9.39	10.88
75-84	14.06	13.06	15.14
85+	20.24	18.79	21.80
**Sex**			
Female	Ref		
Male	1.14	1.13	1.15
**Race**			
Others	Ref		
Indigenous	1.19	1.07	1.32
**COVID-19 Vaccination status**			
Booster	0.58	0.57	0.59
2 doses	Ref		
1 dose	1.32	1.30	1.35
0 doses	1.60	1.58	1.62
**Year**			
2021	Ref		
2022	0.89	0.88	0.90
**Comorbidities**			
Chronical cardiac illness	1.13	1.12	1.14
Hematologic disease	1.18	1.12	1.24
Carrier chromosomal disease immunological fragility	1.57	1.48	1.67
Hepatic disease	0.87	0.84	0.91
Diabetes	0.90	0.88	0.91
Chronical neurological neuromuscular illness	0.77	0.75	0.79
Decompensated chronical respiratory diseases	2.86	2.68	3.05
Immunosuppression	0.72	0.69	0.74
Renal disease	0.65	0.63	0.67
Obesity	0.70	0.68	0.71
Neoplasia	2.71	2.57	2.86
Other	0.99	0.98	1.01

Supplementary Table S4. Multivariate Logistic regression for fatal outcome among COVID-19 confirmed cases in Colombia including reported comorbidities.

Characteristic	Colombia		
	**OR**	**95% CI**	
**Age (years)**			
0-4	1.47	1.14	1.90
5-17	Ref		
18-29	4.60	3.85	5.51
30-39	15.49	13.03	18.43
40-49	44.57	37.54	52.92
50-64	126.02	106.25	149.48
65-74	331.37	279.29	393.16
75-84	728.44	613.72	864.60
85+	1589.08	1337.63	1887.79
**Sex**			
Female	Ref		
Male	1.60	1.57	1.62
**Race**			
Others	Ref		
Indigenous	1.05	0.98	1.13
**COVID-19 Vaccination status**			
2 doses	Ref		
1 dose	6.99	6.81	7.17
0 doses	31.69	31.09	32.29
**Comorbidities**			
Arthritis	2.55	2.35	2.78
Cancer	1.46	1.39	1.53
Diabetes Mellitus	1.82	1.78	1.87
Hypertension	1.73	1.70	1.77
Orphan diseases	2.39	2.14	2.68
HIV infection	2.22	1.96	2.51
**Year**			
2021	Ref		
2022	0.30	0.30	0.31

Supplementary Table S5. Multivariate Logistic regression for fatal outcome among COVID-19 confirmed cases in Mexico including reported comorbidities.

Characteristic	Mexico		
	OR	95% CI	
**Age (years)**			
0-4	1.43	1.23	1.66
5-17	Ref		
18-29	1.24	1.09	1.40
30-39	1.72	1.52	1.93
40-49	2.15	1.91	2.41
50-64	2.40	2.14	2.70
65-74	2.44	2.17	2.74
75-84	2.13	1.89	2.39
85+	1.55	1.37	1.76
**Sex**			
Female	Ref		
Male	1.31	1.28	1.34
**Race**			
Others	Ref		
Indigenous	1.21	1.11	1.33
Not specified	1.33	1.25	1.41
**Comorbidities**			
Diabetes	1.04	1.02	1.07
COPD	0.86	0.80	0.91
Asthma	1.04	0.96	1.13
Immunosuppressed	1.20	1.12	1.29
Hypertension	1.09	1.06	1.11
Cardiovascular disease	1.07	1.01	1.13
Obesity	1.65	1.60	1.69
Chronic renal failure	0.83	0.79	0.87
Other comorbidities	1.05	1.00	1.11
**Year**			
2021	Ref		
2022	0.79	0.77	0.81

Supplementary Figure S1. Data sources and variables across countries. *In Argentina, data were obtained from the COVID-19 surveillance dataset. Brazil's data were sourced from two databases available on the openDATASUS platform of the Brazilian Ministry of Health: (i) the eSUS-Notifica SG database for cases with mild-moderate acute respiratory syndrome, and (ii) the SIVEP-Flu database for cases with severe acute respiratory syndrome. In Colombia, data were retrieved from two modules within the SISPRO database: the COVID module, which contains case-level information on COVID-19 confirmed cases reported by health institutions through the SEGCOVID-19 application, linked with data on mortality from the Unique Registration of Affiliates (Registro Único de Afiliados, RUAF), and information on vaccination status from the PAIweb application. Cases requiring ventilatory support were obtained from the Individual Registry of Health Services Provision module (Registro Individual de Prestación de Servicios de Salud, RIPS), filtered by procedure codes defined in Supplementary Table S1 and the U071 ICD-10 diagnosis code for COVID-19. Due to the lack of linkage between the RIPS database and the COVID-19 module, the comorbidities or vaccination status of cases requiring ventilatory support could not be determined. Information on ICU admissions was not available for Colombia. Finally, for Mexico, we utilized the COVID-19 database consolidated by the Epidemiological Surveillance System for Viral Respiratory Diseases, receiving reports from both the public and private health sectors. Databases can be accessed through the following sources*: [1] http://datos.salud.gob.ar/dataset?groups=covid-19; [2]https://opendatasus.saude.gov.br/;[3]https://www.minsalud.gov.co/proteccionsocial/Paginas/SistemaIntegraldeInformaci%C3%B3nSISPRO.aspx; [4] https://datos.gob.mx/busca/dataset?tags=covid-19

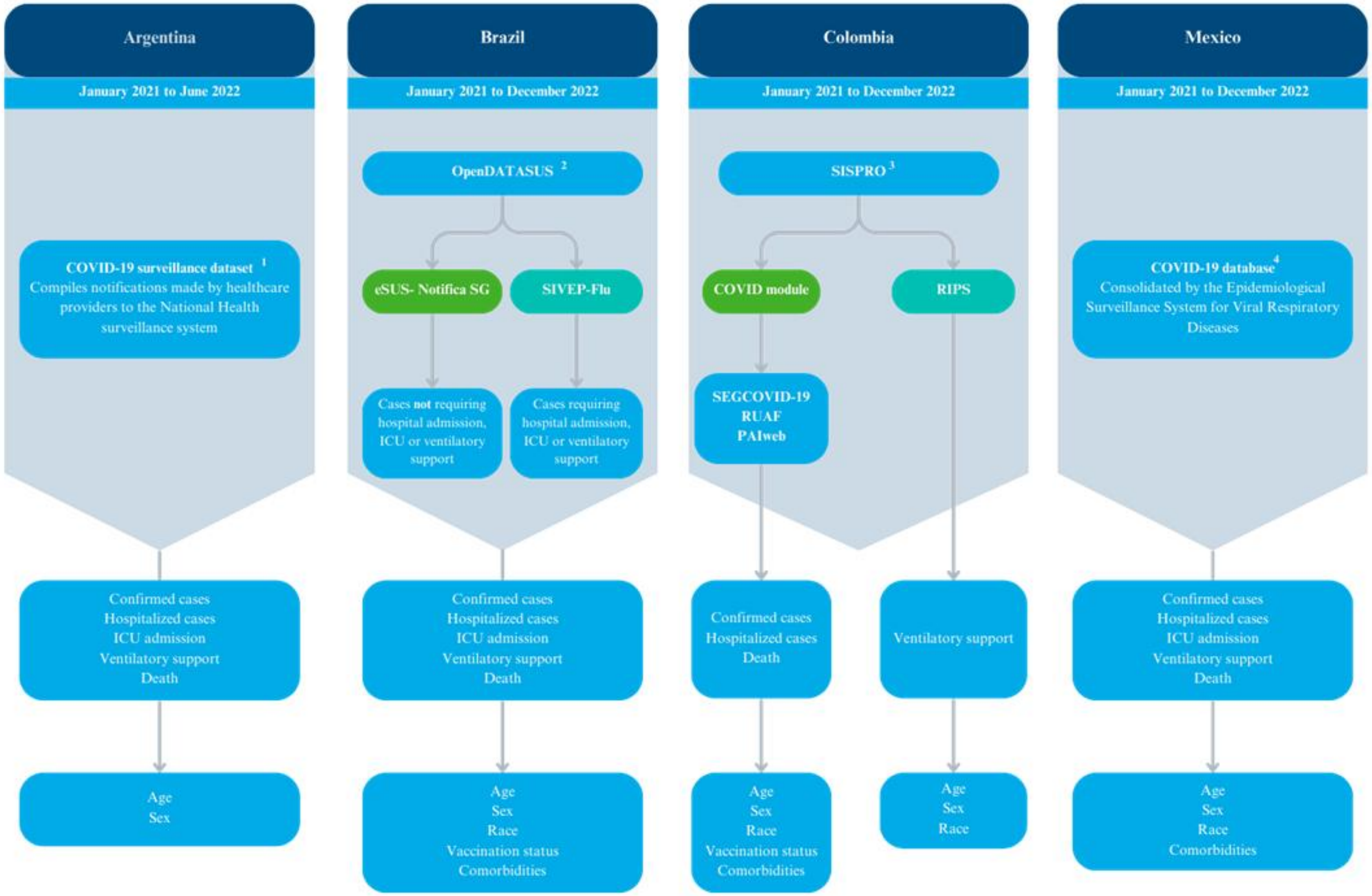

Argentina
January 2021 to June 2022
COVID-19 surveillance dataset [1]
Compiles notifications made by healthcare providers to the National Health surveillance system
Confirmed cases
Hospitalized cases
ICU admission
Ventilatory support
Death
Age
Sex
Brazil
January 2021 to December 2022
OpenDATASUS [2]
eSUS- Notifica SG
SIVEP-Flu
Cases not requiring hospital admission, ICU or ventilatory support
Cases requiring hospital admission, ICU or ventilatory support
Confirmed cases
Hospitalized cases
ICU admission
Ventilatory support
Death
Age
Sex
Race
Vaccination status
Comorbidities
Colombia
January 2021 to December 2022
SISPRO [3]
COVID module
RIPS
SEGCOVID-19
RUAF
PAIweb
Confirmed cases
Hospitalized cases
Death
Ventilatory support
Age
Sex
Race
Vaccination status
Comorbidities
Age
Sex
Race
Mexico
January 2021 to December 2022
COVID-19 database [4]
Consolidated by the Epidemiological Surveillance System for Viral Respiratory Diseases
Confirmed cases
Hospitalized cases
ICU admission
Ventilatory support
Death
Age
Sex
Race
Comorbidities

Supplementary Figure S2. Flowchart of included cases.

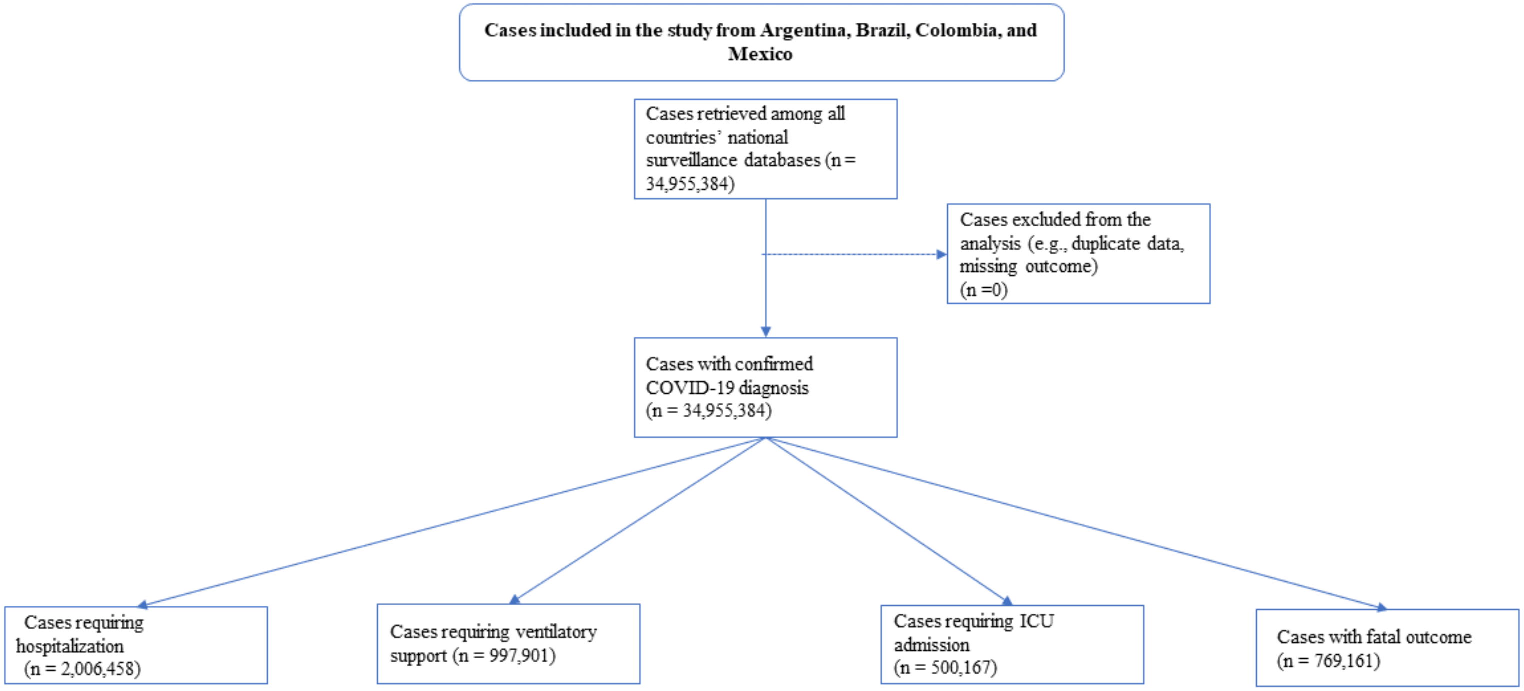

Supplement: Online Supplementary Document [file jogh-15-04141-s001.pdf]
